# Editorial note: Randomized controlled trial of early, small-volume formula supplementation among newborns: a study protocol

**DOI:** 10.1371/journal.pone.0321977

**Published:** 2025-04-01

**Authors:** The PLOS One Editors

After publication of this Study Protocol [1], concerns were raised about undisclosed competing interests and about the ethics approval of the study.

The ethics approval information is reported in the “Ethics approvals and consent” section of [1]. PLOS received documentary verification of all ethics approvals included in the ethics statement.

The Competing Interests statement is updated to:

Dr. Roberts declares receipt of research funding from Danone International for development of a questionnaire assessing food culture in an adult population and is named as a co-inventor on a patent entitled “Compositions and methods for treating and preventing malnutrition” (WO2017120033A1). The authors stated that both of these activities are unrelated to the present project. All other authors have declared that no competing interests exist. The study product will be purchased retail; Abbott Nutrition has no role in the study other than manufacturing the formula for commercial sale.

Results from the pilot randomized controlled trial were published in [2].

The study design of [1] and the ethics of formula milk research in low- and middle-income countries (LMIC) have been discussed in [3–5]. Additional discourse can be found in comments posted on the article webpages for [1] (available from [6]) and [2].
